# Market of First Launch for High-Risk Therapeutic Medical Devices

**DOI:** 10.1001/jamanetworkopen.2024.49298

**Published:** 2024-12-06

**Authors:** Kushal T. Kadakia, Christina Lalani, Daniel B. Kramer, Hibiki Orui, Robert W. Yeh

**Affiliations:** 1 Harvard Medical School, Boston, Massachusetts; 2 Division of Cardiology, Beth Israel Deaconess Medical Center, Harvard Medical School, Boston, Massachusetts; 3 Richard A. and Susan F. Smith Center for Outcomes Research in Cardiology, Division of Cardiology, Beth Israel Deaconess Medical Center, Boston, Massachusetts

## Abstract

This cross-sectional study examines the marketing history of high-risk medical devices launched first in the international market or the US, including specialty, time from US Food and Drug Administration Review to launch, and recall history.

## Introduction

Many medical devices first progress to market outside the US.^1,2^ Over the past decade, Congress and the US Food and Drug Administration (FDA) have implemented policies to expedite domestic device development.^3,4^ We assessed trends in the market of first launch (international vs the US) for high-risk therapeutic medical devices from January 1, 2013, through December 31, 2023.

## Methods

In this cross-sectional study, we identified the market of first launch for all high-risk therapeutic medical devices approved from 2013 to 2023. High-risk devices were defined as class III devices approved under the FDA’s premarket approval (PMA) pathway. Using the PMA database, we collected information on specialty, implantable or life-sustaining status, and special regulatory designations (expedited review, priority review, and breakthrough designation). We excluded devices with diagnostic indications, previous FDA authorizations, and missing materials (eMethods in Supplement 1). To determine the market of first launch, we reviewed each device’s summary of safety and effectiveness data (SSED), including a disclosure of marketing history for the device, which the FDA must publish by law.^5^ We documented whether the device was authorized outside the US and subject to any market withdrawals prior to FDA approval based on documentation in the marketing history section of the SSED. For devices with prior international approvals, we determined the time until FDA approval. To evaluate changes in market launch patterns over time, we performed χ^2^ tests for linear trend with 2-sided *P* < .05 considered statistically significant.

All analyses used R, version 4.4.0. The study was exempted from review by the Institutional Review Board of Harvard Medical School due to its use of publicly available data and followed the STROBE reporting guidelines.

## Results

From 2013 through 2023, the FDA approved 379 high-risk therapeutic devices through the PMA pathway, of which 230 met inclusion criteria (Table). The most common specialties were cardiovascular (114 [49.6%]), orthopedic (24 [10.4%]), and gastroenterology or urology (19 [8.3%]). Most devices were implantable (160 [69.6%]), and 65 (28.3%) were designated as life sustaining or supporting. Thirty-three devices (14.3%) carried a special regulatory designation, and 19 (8.3%) were designated breakthrough devices. Median FDA review time was 0.96 (IQR, 0.62-1.56) years for all devices, and 0.65 (IQR, 0.49-1.03) years for devices with special regulatory designations.

**Table.** Characteristics of High-Risk Therapeutic Devices Approved by the FDA, 2013 to 2023

Characteristic	Devices		
	All (N = 230)	First launch	
		International (n = 184)	US (n = 46)
Regulatory, No. (%)			
Specialty			
Cardiovascular	114 (49.6)	93 (50.5)	21 (45.7)
Orthopedic	24 (10.4)	23 (12.5)	1 (2.2)
Ophthalmic	17 (7.4)	12 (6.5)	5 (10.9)
Gastroenterology or urology	19 (8.3)	16 (8.7)	3 (6.5)
Neurology	17 (7.4)	12 (6.5)	5 (10.9)
Implantable device designation	160 (69.6)	137 (74.5)	23 (50.0)
Life sustaining or supporting designation	65 (28.3)	57 (31.0)	8 (17.4)
Special regulatory designation	33 (14.3)	24 (13.0)	9 (19.6)
Breakthrough devices	19 (8.3)	12 (6.5)	7 (15.2)
Time to market launch, median (IQR), y			
FDA review	0.96 (0.62-1.56)	0.97 (0.63-1.52)	0.95 (0.62-1.56)
FDA review for devices with special regulatory designations	0.65 (0.49-1.03)	0.81 (0.55-1.19)	0.49 (0.47-0.65)
Time elapsed between international and FDA approval^a^	5.1 (3.4-9.0)	NA	NA
Time elapsed for devices with special regulatory designations	5.7 (3.6-10.4)	NA	NA
Safety signals, No. (%)			
Market withdrawals prior to FDA approval	8 (3.5)	8 (4.3)	NA
Recalls following FDA approval	67 (29.1)	58 (31.5)	9 (19.6)
Class I FDA recalls	20 (8.7)	17 (9.2)	3 (6.5)

Abbreviations: FDA, US Food and Drug Administration; NA, not applicable.

^a^ Based on 121 of 184 devices with international-first launches that specified the date of previous international approval in FDA’s public database.

The US was the market of first launch for 46 devices (20.0%), with 9 (19.6%) carrying special regulatory designations. There was a statistically significant increase in the proportion of US-first launches from 2013 to 2023 (from 2 devices to 12; *P* = .001 for trend) (Figure). International approval dates were available for 121 of the 184 devices first launched outside the US. For these, the median time elapsed between international and FDA approval was 5.1 (IQR, 3.4-9.0) years for all devices and 5.7 (IQR, 3.6-10.4) years for devices with special regulatory designations. Eight of 184 devices (4.3%) first launched internationally had a market withdrawal prior to FDA approval. Following FDA approval, 67 of the 230 devices (29.1%) were recalled (including 9 of 46 [19.6%] US-first devices), with 20 (8.7%) recalls designated as class I (most severe, including 3 of 46 US-first devices [6.5%]).

**Figure.**
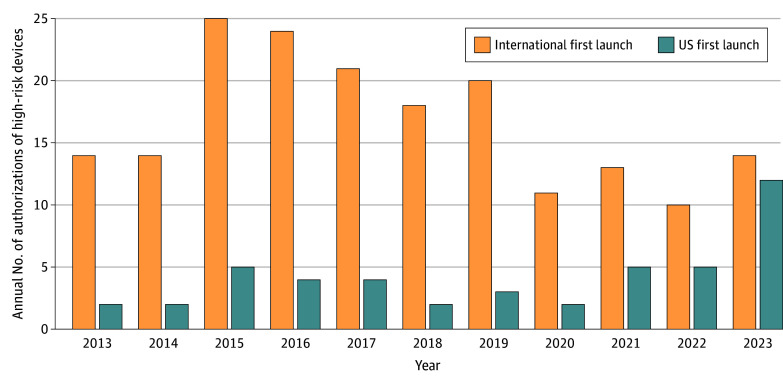
Trends in US-First Launches of High-Risk Therapeutic Devices Using the χ^2^ test for linear trend, *P* = .001 for US- vs international-first launches of devices.

## Discussion

While many high-risk therapeutic devices continue to be launched internationally, the proportion of US-first launches has increased since 2013. One in 5 US-first approvals is designated for expedited review, reflecting potential contribution of recent regulatory reforms.

Study limitations include exclusion of diagnostic and lower-risk devices and missing international authorization dates. Although earlier access can offer benefits, previous studies have identified greater safety risks for US-first devices.^1,2,6^ Consequently, policy makers should ensure that evolving trends in device innovation are paired with adequate requirements for evidence generation across the device life cycle.

## Notes

Supplement 1. **eMethods.** Sample Construction and Comparison of Launch Terms

Supplement 2.
Data Sharing Statement
