# Supplementary material for: Market of First Launch for High-Risk Therapeutic Medical Devices
Source: JAMA Netw Open. 2024 Dec 6;7(12):e2449298. doi: 10.1001/jamanetworkopen.2024.49298 (PMC11624582; doi:10.1001/jamanetworkopen.2024.49298)
Supplement: Supplement 1. — eMethods. Sample Construction and Comparison of Launch Terms [file jamanetwopen-e2449298-s001.pdf]

Supplementary Online Content

Kadakia KT, Lalani C, Kramer DB, Orui H, Yeh RW. Market of first launch for high-risk therapeutic medical devices. *JAMA Netw Open*. 2024;7(12):e2449298.
doi:10.1001/jamanetworkopen.2024.49298

eMethods. Sample Construction and Comparison of Launch Terms

This supplementary material has been provided by the authors to give readers additional information about their work.

© 2024 Kadakia KT et al. *JAMA Network Open*.

eMethods. Sample Construction and Comparison of Launch Terms

Sample Construction

Selection of FDA Review Pathway

The FDA has multiple review pathways for medical devices. We focused on devices undergoing review in the Premarket Approval (PMA) pathway, as this pathway is reserved for the highest-risk, Class III, devices and requires clinical evidence of safety and effectiveness. Within the PMA pathway, there are "Original" PMA submissions (which reflect the initial authorization for a device in the U.S. market) and "Supplemental" PMA submissions (which reflect changes affecting the safety or effectiveness of already approved devices). Given our focus on launch times for novel devices, we focused on the "Original" submissions.

Exclusion Criteria

To be included in the final cohort, devices needed to:

1. Have publicly available copies of their Summary of Safety and Effectiveness Data (SSED) memos available in FDA's PMA database.
2. Not have previous authorizations under a different FDA review pathway prior to their approval under the PMA pathway. This exclusion criteria did not apply to devices that (1) were applying for a new, higher-risk level of indication for use, and (2) devices that had provisionally been authorized for use under the Humanitarian Device Exemption, but had not received full FDA approval for commercial marketing without restrictions on use.
3. Have a therapeutic indication for use.

FDA's PMA database contained 379 Original approvals from 2013-2023.

- For 23 PMAs, no SSED was available; 20 of these missing submissions were for Biologics approvals, while 3 were for cardiac ablation devices. *Example: P210027 was approved by the FDA in 2022. However, FDA's database only includes an Approval Order statement, not the full SSED. Therefore, it was excluded.*
- For 20 PMAs, the PMA submissions were due to reclassifications of devices that had been previously authorized under other FDA pathways (e.g., 510(k)) rather than a truly de novo PMA submission. *Example: P160015 was first authorized for use in the U.S. by FDA under the 510(k) pathway in 2002. It was reclassified under a change in FDA regulations in 2015, which required resubmission of the device as a PMA, with its new approval granted by FDA in 2017. Therefore, it was excluded.*
- Of the remaining 336 PMA devices, 106 devices had diagnostic indications for use. Diagnostic devices were excluded as products are frequently "re-launched" in different countries due to changes in manufacturing processes as opposed to intrinsic changes in device design.

After excluding these 149 devices, 230 devices remained that met inclusion criteria.

Comparison of Launch Times

If the SSED "Marketing History" section indicated that the device had previously been authorized internationally, then we used listed date of international marketing as a point of comparison.

Caveats

- If a device's SSED did not provide a specific date for the previous international authorizations, then the device was not included for calculations of launch time comparisons. This issue affected 63 devices in our sample. *Example: P220023 was authorized in over 50 countries prior to gaining FDA approval. However, the SSED does not provide a date for when the device first became available internationally.*
- If a device's SSED included multiple dates of international authorization for different countries, then we used the earliest date of availability. *Example: P200029 was first marketed in Canada in 2005, then Europe in 2006, then MENA in 2009, then Asia in 2015, and Latin America in 2018. Since 2005 was the first date of international availability, we used this for our analysis*.
- If a device had been modified following its initial authorization, we used the earliest date of authorization for the device that most closely resembled the version of the device authorized in the U.S. *Example: P140012 is a device with multiple components including both a balloon and a delivery catheter. The balloon component was authorized in Europe in 2007. The catheter component was authorized in Europe in 2010. Since the final component of the device was authorized in 2010, we used this latter date as the date of "launch."*
- If a device's SSED includes a date for international authorization that was limited to only the year, then we determined the midpoint of the year for the purpose of calculating the time elapsed between international approval and FDA approval. *Example: P220024 received FDA approval on June 2, 2023. According to its SSED, it has been available outside of the US since 2014. Therefore, for the purposes of calculation, it was assigned a midpoint date of July 2, 2014, with the time elapsed between international approval and FDA approval determined to be 8.9 years*.

© 2024 Kadakia KT et al. *JAMA Network Open*.
