# Supplementary material for: Market of First Launch for High-Risk Therapeutic Medical Devices
Source: JAMA Netw Open. 2024 Dec 6;7(12):e2449298. doi: 10.1001/jamanetworkopen.2024.49298 (PMC11624582; doi:10.1001/jamanetworkopen.2024.49298)
Supplement: Supplement 2. — Data Sharing Statement [file jamanetwopen-e2449298-s002.pdf]

Data Sharing Statement

Kadakia. Market of First Launch for High-Risk Therapeutic Medical Devices. *JAMA Netw Open*. Published December 06, 2024. doi:10.1001/jamanetworkopen.2024.49298

Data
Data available: Yes
Data types: Data (not involving human participants)
How to access data: Requests can be sent to ryeh@bidmc.harvard.edu
When available: With publication

Supporting Documents
Document types: None

Additional Information
Who can access the data: Researchers with approved proposal
Types of analyses: Research
Mechanisms of data availability: After approval of proposal.
